# Exploring prognostic genes related to lactylation and programmed cell death in pancreatic ductal adenocarcinoma: a comprehensive study combining bulk transcriptomics and experimental verification

**DOI:** 10.3389/fgene.2026.1774953

**Published:** 2026-03-12

**Authors:** Boxing Zhang, Gen Sun, Luying Huang, Wenjun Wei, Yuzhang Yuan, Zehua Wang, Xingzhou Peng, Liang Song, Kıvanç Görgülü, Jiaoyu Ai

**Affiliations:** 1 School of Clinical Medicine, Shaanxi University of Chinese Medicine, Xianyang, China; 2 Department of General Surgery, The First Affiliated Hospital, Jiangxi Medical College, Nanchang University, Nanchang, China; 3 Department of Gastroenterology, The First Affiliated Hospital, Jiangxi Medical College, Nanchang University, Nanchang, China; 4 Department of Oncology, The First Affiliated Hospital of Zhengzhou University, Zhengzhou, China; 5 School of Biomedical Engineering, Hainan University, Sanya, China; 6 Medical Experiment Center, Shaanxi University of Chinese Medicine, Xianyang, China; 7 Institute for Tumor Metabolism, Comprehensive Cancer Center München, TUM School of Medicine and Health, University Medical Center, Technical University of Munich, Munich, Germany; 8 Institute of Metabolism and Cell Death, Helmholtz Zentrum München, Munich, Germany

**Keywords:** immune microenvironment, lactylation, pancreatic ductal adenocarcinoma, prognostic risk model, programmed cell death

## Abstract

**Background:**

There is a strong correlation between lactylation, programmed cell death, and the progression of cancer. This study aims to identify prognostic genes associated with lactylation and programmed cell death in pancreatic ductal adenocarcinoma (PDAC), providing new insights for risk stratification and therapeutic strategies.

**Methods:**

TCGA-PAAD, GSE62452, lactylation-related genes (LRGs), and programmed cell death-related genes (PCDRGs) were retrieved from relevant databases and references. Prognostic genes were identified through univariate Cox regression analysis, followed by random survival forest analysis for survival prediction. Subsequently, enrichment analysis, immune microenvironment analysis, drug sensitivity analysis, immunohistochemical analysis, and expression analysis of prognostic genes were conducted. Finally, the experimental verification was carried out in clinical samples.

**Results:**

In this investigation, two prognostic genes (HMGA1 and KIF2C) linked to lactylation and programmed cell death were identified, and a robust prognostic risk model was developed. Enrichment analysis results included Cell cycle, G2M checkpoint, Myogenesis, and Angiogenesis. Moreover, immature B cells and activated B cells demonstrated the strongest positive correlation (cor = 0.97, *P* < 0.001), while neutrophils and activated B cells demonstrated the strongest negative correlation (cor = −0.68, *P* < 0.001). Furthermore, KIF2C and HMGA1 demonstrated the strongest negative relationships with mast cells (correlation coefficients = −0.36 and −0.53, *P* < 0.01). Drug sensitivity analysis revealed that Sapitinib was more effective in the high-risk group (HRG), while Doramapimod was more effective in the low-risk group (LRG) (*P* < 0.0001). Both immunohistochemical and expression analyses of prognostic genes showed that HMGA1 and KIF2C were upregulated in PDAC patients (*P* < 0.05). Finally, genes in the clinical samples also showed the same expression trend.

**Conclusion:**

In the present investigation, two prognostic genes were identified, and subsequently, a predictive risk model was established, which may serve as a valuable reference for the clinical management of PDAC.

## Graphical Abstract

**Figure:**
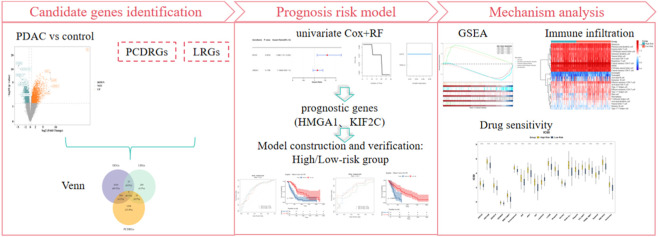

## 1 Introduction

Pancreatic ductal adenocarcinoma (PDAC) accounts for approximately 85% of all pancreatic cancer cases and is one of the lethal malignancies with an extremely poor prognosis (Jentzsch et al., 2020; Narayanan et al., 2021). Its 5-year overall survival rate remains low at about 9%–11%, making it a major cause of cancer-related death (Mizrahi et al., 2020; Hsu et al., 2022). Alarmingly, while the overall cancer mortality rate has decreased, the incidence of PDAC continues to rise (Siegel et al., 2024), with predictions showing that by 2030–2040, it will become the second leading cause of cancer death after lung cancer (Rahib et al., 2014; Rahib et al., 2021). The poor prognosis of PDAC is mainly attributed to early systemic spread, invasive local infiltration, and limited therapeutic efficacy (Connor and Gallinger, 2022; Garajová et al., 2023). The disease typically progresses through multiple stages from precancerous lesions to low-grade and high-grade dysplasia, characterized by progressive cytological atypia and genetic aberrations. Clinical manifestations often include non-specific symptoms such as loss of appetite, nausea, indigestion, back pain, and unexplained weight loss, while jaundice frequently appears as a late indicator. Although surgical resection remains the most effective treatment modality, only about 20% of patients meet the criteria for curative surgery at the time of diagnosis (Schneider et al., 2021; Garajová et al., 2023). Therefore, identifying new prognostic biomarkers is crucial for understanding the molecular mechanisms of PDAC and realizing individualized treatment strategies.

Lactate, as one of the most abundant metabolites in the circulatory system, is produced through the action of lactate dehydrogenase (LDH) on pyruvate during glycolysis (Rabinowitz and Enerbäck, 2020). Under hypoxic conditions, pyruvate is converted to lactate, allowing for the regeneration of NAD+, which is crucial for maintaining glycolytic flux (Li L. et al., 2023). Notably, even under normoxic conditions, cancer cells preferentially metabolize glucose into lactate—a phenomenon known as the Warburg effect—leading to abnormal protein lactylation modifications in the tumor microenvironment (Wang and Patti, 2023; Yu et al., 2024). Lactylation, a newly discovered post-translational modification, has emerged as a key regulator of gene expression and cell metabolism during cancer progression. In PDAC, the accumulation of lactate within the tumor microenvironment has been proven to drive histone lactylation both *in vitro* and *in vivo*, potentially affecting tumor cell behavior and immune evasion (Li F. et al., 2024).

Programmed cell death (PCD) encompasses multiple genetically regulated pathways, such as apoptosis, autophagy, ferroptosis, pyroptosis, and necroptosis. These five represent some of the 18 characterized forms. In PDAC, tumor cells exhibit significant differences in ferroptosis sensitivity, with some cells escaping ferroptosis by upregulating antioxidant systems. This suggests that inducing ferroptosis could be a novel therapeutic strategy (Li G. et al., 2024). Interestingly, lactate plays a dual role in cell death: it can inhibit apoptosis by creating an acidic microenvironment, thereby enhancing tumor cell survival, while, under specific conditions, lactate accumulation may paradoxically induce ferroptosis (Zhao et al., 2020; Yang Z. et al., 2023). Despite these emerging findings, the mechanistic interaction between lactylation and programmed cell death in PDAC remains incompletely elucidated, and no research has yet established a prognostic model for PDAC that integrates these two biological processes.

This study used public PDAC datasets to identify differentially expressed genes associated with lactylation and programmed cell death via differential expression analysis. Prognostic genes were identified using univariate and random forest algorithms, followed by the construction and validation of a predictive risk model. Clinical feature analysis, functional enrichment analysis, immune correlation analysis, tumor mutation burden, and drug sensitivity analysis were also performed. Additionally, the prognostic genes were subjected to chromosomal localization and immunohistochemistry analysis. Finally, experimental verification confirmed that the expression of prognostic genes was consistent with the bioinformatics results. This study aims to provide a new theoretical basis for the clinical prognostic evaluation and the formulation of treatment strategies for PDAC.

## 2 Materials and methods

### 2.1 Data collection

The training set of PDAC patients was derived from the Cancer Genome Atlas (TCGA) database (https://portal.gdc.cancer.gov/), including 143 tumor (PDAC) and four para-cancerous control tissue samples. Of these, 142 patients had survival information. Meanwhile, the clinical data and somatic mutation data were also collected. The validation set GSE62452 (GPL6244), which encompassed tumor tissue specimens from 65 PDAC patients with survival information, was derived from the Gene Expression Omnibus (GEO) database (http://www.ncbi.nlm.nih.gov/geo/). The visit took place on 26 February 2025. Furthermore, a total of 332 lactylation-related genes (LRGs) were obtained from reference (Huang et al., 2023) (Supplementary Table S1), and 1,548 programmed cell death-related genes (PCDRGs) were obtained from reference (Qin et al., 2023) (Supplementary Table S2).

### 2.2 Differential expression analysis

To identify differentially expressed genes (DEGs) between PDAC and control samples within the training set, the “DESeq2” package (v 1.38.0) (Love et al., 2014) was employed for differential expression analysis, and DEGs were screened based on *P* < 0.05 and |log_2_ fold change (FC)| >0.5. Subsequently, the “ggplot2” package (v 3.4.1) (Gustavsson et al., 2022) was utilized to construct a volcano plot for visualizing the DEGs.

### 2.3 Identification and functional characterization of candidate genes

To identify the DEGs linked to lactylation and programmed cell death in PDAC, the “ggvenn” package (v 0.1.9) (Chen and Boutros, 2011) was used to intersect DEGs, LRGs, and PCDRGs; the intersection genes were used as candidate genes. Next, to explore the functional pathways related to candidate genes in PDAC, gene set enrichment analysis (GSEA) was performed. The reference gene set “c2.cp.kegg.v7.5.1.symbols” was carefully chosen from the Molecular Signatures Database (MSigDB, https://www.gsea-msigdb.org/gsea/msigdb). First, based on all samples in the training set, Spearman correlation analyses were performed between each candidate gene and all the remaining genes in the training set, separately, using the “psych” package (v 2.2.9) (Orifjon et al., 2023) to obtain the correlation coefficients for all samples in the training set. Subsequently, genes were arranged in descending order according to these coefficients. The sorted data were then utilized to conduct GSEA (|normalized enrichment score (NES)| > 1 and *P* < 0.05) using the implementation of the “clusterProfiler” package (v 4.2.2) (Yu et al., 2012).

### 2.4 Determination of prognostic genes, development, and validation of a prognostic risk model

Within the samples of PDAC with survival information in the training set, univariate Cox regression analysis was utilized to analyze the candidate genes by the “survival” package (v 3.5.3) (Kumar et al., 2020) (hazard ratio (HR) ≠ 1, *P* < 0.2). Then, Random Survival Forest (RSF) analysis was conducted for the outcomes of the proportional hazards (PH) assumption test (*P* > 0.05) using the “randomForestSRc” package (v 3.2.3) (Ishwaran et al., 2008) to obtain risk scores for each patient.

Additionally, patients were categorized into a high-risk group (HRG) and a low-risk group (LRG) based on the optimal threshold derived from PDAC samples with survival information. The “survival” package (v 3.5.3) (Kumar et al., 2020) was used to plot the Kaplan-Meier (K-M) survival curve (*P* < 0.05, log-rank test). Furthermore, the risk score distribution maps and survival state distribution maps were plotted using the “ggplot2” package (v 3.4.1) (Gustavsson et al., 2022). Next, the receiver operating characteristic (ROC) curve of the prognostic risk model was plotted using the “survivalROC” package (v 1.0.3.1) (Zheng et al., 2021). The area under the curve (AUC) was calculated to evaluate the model’s effectiveness (AUC >0.6). Finally, the model’s reliability was validated in the validation set using the above method.

### 2.5 Clinicopathological characteristics analysis

Among the samples with survival information in the training set, the “pheatmap” package (v 1.0.12) (Wang et al., 2023c) was used to draw a heat map to show the distribution of risk scores in clinicopathological characteristics (age, gender, stage T, N, and tumor stage) and the expression of prognostic genes in HRG and LRG.

### 2.6 Gene set enrichment analysis (GSEA) and gene set variation analysis (GSVA)

To examine the biological functions and pathways among patients in distinct risk groups, the following analysis was conducted. The “c2.cp.kegg.v7.5.1.symbols.gmt” gene set was acquired from the MSigDB as the reference gene set. Within the samples of PDAC with survival information in the training set, the “DESeq2” package (v 1.38.0) (Love et al., 2014) was utilized to scrutinize the disparities between HRG and LRG to obtain the corresponding genes and log_2_FC values. Subsequently, the genes were arranged in descending order by log_2_ fold change log_2_FC. The “clusterProfiler” package (v 4.2.2) (Yu et al., 2012) was utilized to carry out GSEA (|NES| >1 and *P* < 0.05).

GSVA was conducted in HRG and LRG using the “GSVA” package (v 1.46.0) (Hänzelmann et al., 2013). The “h.all.v7.5.1.symbols.gmt” gene set was obtained from the MSigDB as the reference gene set. The ssGSEA scores of the signaling pathways were evaluated. The “limma” package (v 3.58.1) (Ritchie et al., 2015) was utilized to compare the differences in scores between groups. Generally, |t| >2 and *P* < 0.05 were considered to indicate a significant difference.

To quantify lactylation activity, a score was calculated via ssGSEA using a curated gene set (including LDHA/B, SIRT1/2, EP300, and GTPSCS) and compared between risk groups. This activity score was then correlated with HMGA1 and KIF2C expression to evaluate their relationship with the tumor’s lactylation-associated metabolic landscape.

### 2.7 Analysis of immune microenvironment

To understand the immune infiltration of different samples in the training set, the ssGSEA algorithm was employed to analyze the distribution of 28 distinct immune cells (Jiang et al., 2022) in both HRG and LRG samples. Subsequently, the Wilcoxon test was harnessed to investigate the disparities in the infiltration levels of these 28 immune cells between HRG and LRG samples (*P* < 0.05), and the results were visualized using the “ggplot2” package (v 3.4.1) (Gustavsson et al., 2022). Furthermore, Spearman correlation was performed to investigate the connections between the differential immune cells, as well as differential immune cells and prognostic genes (|correlation coefficient (cor)| >0.3 and *P* < 0.05).

Additionally, to fully understand the immune infiltration landscape of PDAC, the stromal score, immune score, and ESTIMATE score were computed using the “estimate” package (v 1.0.13) (Wang Y. et al., 2022) in the training set, which included tumor samples with survival information. And differences in scores between HRG and LRG were assessed by the Wilcoxon test, with *P* < 0.05. Moreover, Spearman correlations were carried out to investigate the relationships between the risk scores and ESTIMATE scores, immune scores, and stromal scores (|cor| > 0.3 and *P* < 0.05) using the “psych” package (v 2.2.9) (Orifjon et al., 2023).

### 2.8 Drug susceptibility prediction and gene mutation landscape analysis

In the tumor samples with survival information in the training set, PDAC chemotherapeutic agents were retrieved from the Genomics of Drug Sensitivity in Cancer (GDSC) database (https://www.cancerrxgene.org). Moreover, the half-maximal inhibitory concentration (IC_50_) values for the HRG and LRG were estimated using the “pRRophetic” package (v 0.5) (Wang et al., 2023b) to evaluate the drug susceptibility. The sensitivities of the HRG and LRG were contrasted by means of the Wilcoxon test, with *P* < 0.05.

Furthermore, the mutation frequencies of genes in HRG and LRG were analyzed using the “maftools” package (v 2.14.0) (Mayakonda et al., 2018), and the top 20 mutation frequencies were visualized using a waterfall plot. Based on the somatic mutation numbers of PDAC patients, the “maftools” package (v 2.14.0) (Mayakonda et al., 2018) was utilized to calculate the tumor mutational burden (TMB) scores of the patients. The Wilcoxon test was employed to contrast the disparities in TMB scores between the HRG and LRG (*P* < 0.05). Meanwhile, Spearman correlation was performed to investigate the relationship between the risk and TMB scores (|cor| >0.3 and *P* < 0.05) using the “psych” package (v 2.2.9) (Orifjon et al., 2023).

### 2.9 Prognostic gene analysis

To map the positions of prognostic genes on chromosomes, the “RCircos” package (v 1.2.2) (Hu et al., 2014) was implemented.

To study the expression distribution of proteins encoded by prognostic genes in tumor and control tissues, immunohistochemical analysis of prognostic genes in PDAC tissues and control tissues was performed using the Human Protein Atlas (HPA) database (https://www.proteinatlas.org). Additionally, the expression variations of prognostic genes were explored using the Wilcoxon test in PDAC and control samples from the training set (*P* < 0.05).

### 2.10 Reverse transcription quantitative PCR (RT-qPCR)

A total of six PDAC tissues and six control tissue specimens were collected. The six pairs of control and PDAC samples were obtained from the clinic at the First Affiliated Hospital of Nanchang University. The study was approved by the Ethics Committee with Ethical Number 2022 (4–027). RNA concentration was detected using the NanoPhotometer N50, and reverse transcription was performed using a cDNA synthesis kit (HP All-in-one qRT Master Mix II RT203-Ver.1), and primers were synthesized by a biological company (Table 1). The RT-qPCR experiments were performed with GAPDH as the internal reference gene, and the expression levels of the prognostic genes were calculated using the 2^−ΔΔCT^ method. “Graphpad Prism” (v 10.1.2) (Al-Rawi et al., 2023) was employed to plot and calculate the *P* value. Differences between PCR experimental categories were obtained through a t-test (*P* < 0.05).

**TABLE 1:** Primer Sequence.

Primer	Sequence
HMGA1 F	GCA​TCC​GCA​TTT​GCT​ACC​AG
HMGA1 R	TCT​CAG​TGC​CGT​CCT​TTT​CC
KIF2C F	TCC​GTG​TCA​GCC​ATC​AAG​AG
KIF2C R	CAG​GCA​AAC​AGT​CGG​GTA​CT
Internal reference-GAPDH F	ATG​GGC​AGC​CGT​TAG​GAA​AG
Internal reference-GAPDH R	AGG​AAA​AGC​ATC​ACC​CGG​AG

### 2.11 Statistical analysis

Bioinformatics analyses were conducted using the R programming language (v 4.2.2). The disparities between the two groups were evaluated via the Wilcoxon test, with a significance level of *P* < 0.05 considered statistically significant.

## 3 Results

### 3.1 There were five candidate genes affecting the development of PDAC in different ways

**FIGURE 1:**
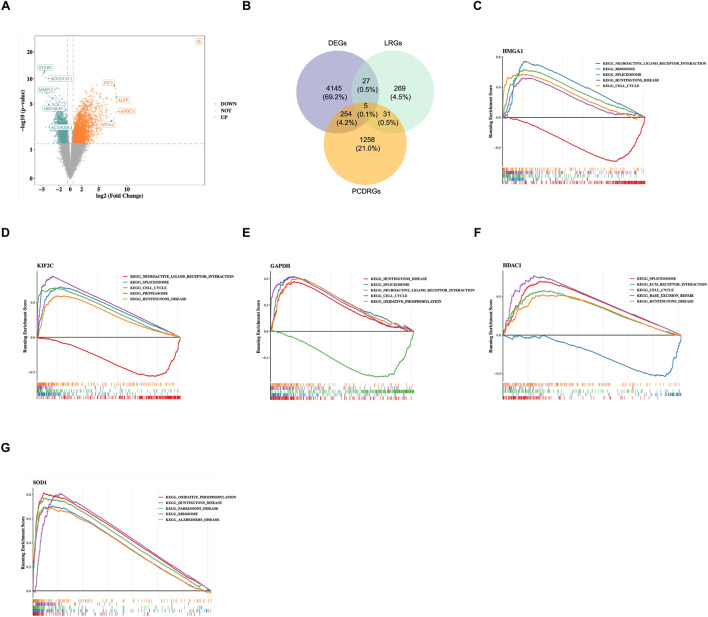
Identification of candidate genes and functional enrichment analysis. **(A)** Volcano plot showing differentially expressed genes (DEGs) between PDAC tumor samples and control samples in the training set. Red dots represent upregulated genes, blue dots represent downregulated genes, and gray dots represent genes with no significant difference. **(B)** Venn diagram showing the intersection of DEGs, lactylation-related genes (LRGs), and programmed cell death-related genes (PCDRGs). **(C–G)** Gene Set Enrichment Analysis (GSEA) results for the five candidate genes. The top five enriched KEGG pathways are shown for HMGA1 **(C)**, KIF2C **(D)**, GAPDH **(E)**, HDAC1 **(F)**, and SOD1 **(G)**.

RGs were intersected, and five intersection genes were obtained as candidate gene. There were 4,431 DEGs between the tumor and control samples in the training set, including 2,363 upregulated genes and 2,068 downregulated genes (Figure 1A). Furthermore, to identify DEGs associated with lactylation and programmed cell death, 4,431 DEGs, 332 LRGs, and 1,548 PCDenes (HMGA1, KIF2C, GAPDH, HDAC1, and SOD1) (Figure 1B). Subsequently, GSEA was carried out for candidate genes. Functional enrichment analysis revealed that HMGA1 (87 pathways), KIF2C (102), GAPDH (101), HDAC1 (93), and SOD1 (114) are involved in diverse biological processes (Figures 1C–G; Supplementary Tables S3–S7). Notably, HMGA1 and KIF2C shared enrichment in 69 pathways, and several lactate metabolism–related pathways, such as KEGG_GLYCOLYSIS_GLUCONEOGENESIS and KEGG_GLYCEROPHOSPHOLIPID_METABOLISM, were significantly upregulated. To clarify the biological relevance of GAPDH and SOD1 in the lactylation-associated context, we found that GAPDH showed a strong positive correlation with LDHA (r = 0.73), the key enzyme for lactate production (Supplementary Figure S1A) Furthermore, single-gene GSEA confirmed that both GAPDH and SOD1 are significantly enriched in lactylation-related metabolic pathways (Supplementary Figure S1B). Overall, the comprehensive analysis has offered a wealth of information that could guide further research efforts aimed at deciphering the underlying mechanisms of PDAC and devising innovative therapeutic approaches.

### 3.2 A total of two genes that impact PDAC prognosis were discovered

**FIGURE 2:**
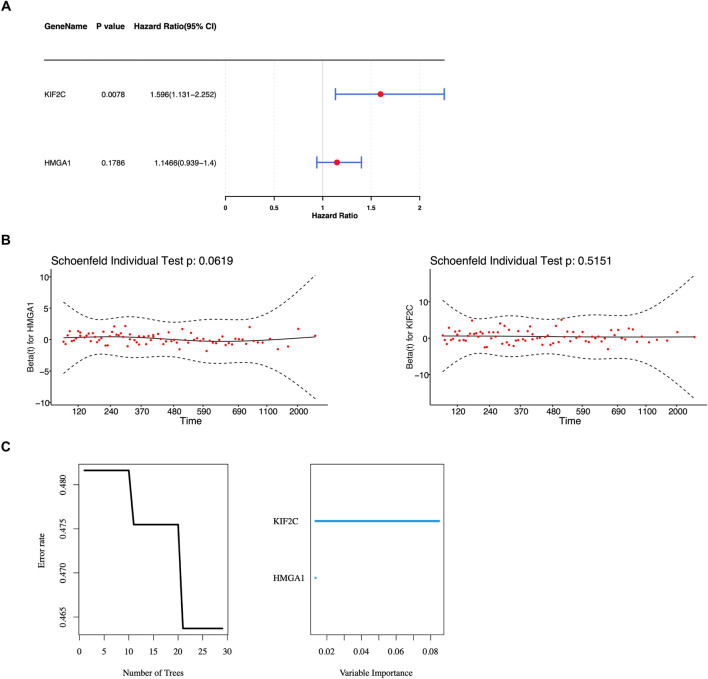
Identification of prognostic genes and construction of a Random Survival Forest model. **(A)** Forest plot showing univariate Cox regression analysis results for the five candidate genes. **(B)** Proportional hazards (PH) assumption test results for HMGA1 and KIF2C. **(C)** Random Survival Forest (RSF) model optimization plot showing the relationship between the number of trees (ntree) and prediction error rate.

Univariate Cox regression analysis was executed based on five candidate genes. Altogether, two genes were obtained (HR ≠ 1, *P* < 0.2) (Figure 2A) (Emura et al., 2019). Next, these two genes (HMGA1 and KIF2C) successfully satisfied the criteria of the PH hypothesis test (*P* > 0.05) (Figure 2B). Subsequently, the RSF model was constructed based on the above two prognostic genes to obtain the risk scores for each patient. It could be seen from the residual plot that when ntree was 29, the error rate of the model was the lowest; therefore, the parameters of the model were set as ntree = 29 and maximum split node mtry = 5 (Figure 2C).

### 3.3 A reliable prognostic risk model was established

**FIGURE 3:**
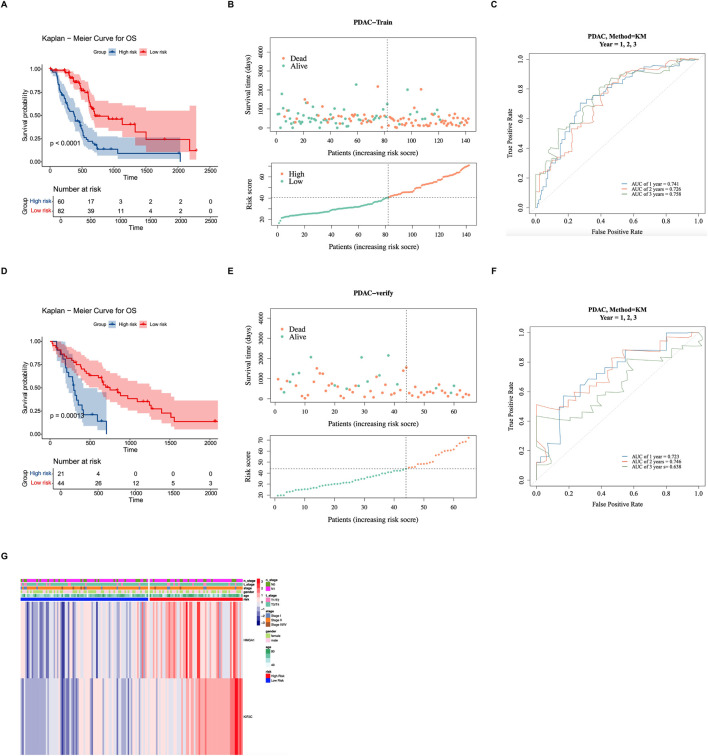
Construction and validation of the prognostic risk model. **(A)** Kaplan-Meier survival curves comparing the high-risk group (HRG) and low-risk group (LRG) in the training set. **(B)** Risk score distribution (upper panel), survival time distribution (middle panel), and survival status (lower panel) of patients in the training set. Patients are ordered by increasing risk score. As risk scores increased, survival time decreased, and mortality rate increased. **(C)** Time-dependent receiver operating characteristic (ROC) curves for the prognostic model in the training set. The area under the curve (AUC) values for 1-, 2-, and 3-year overall survival were 0.741, 0.726, and 0.758, respectively, indicating good predictive performance. **(D–F)** Validation of the prognostic model in the GSE62452 validation set. Kaplan-Meier curves **(D)**, risk score and survival status distribution **(E)**, and time-dependent ROC curves **(F)** confirmed the robustness of the model (*P* = 0.00013; AUC: 0.723, 0.746, and 0.638 for 1-, 2-, and 3-year survival). **(G)** Heatmap showing the distribution of clinicopathological features and prognostic gene expression across risk groups. HMGA1 and KIF2C showed higher expression in the HRG compared to the LRG.

Within the tumor samples with survival information in the training set, patients were segmented into HRG (n = 60) and LRG (n = 82) based on the optimal threshold value (40.52855). Then it was found that there were substantial disparities in survival probability between HRG and LRG (*P* < 0.0001) (Figure 3A); as risk scores increased, survival time decreased, and more deaths occurred (Figure 3B). The AUC value (1-(0.741), 2-(0.726), and 3-(0.758) years) indicated that this prognostic risk model functioned effectively in predicting the survival status (Figure 3C). Then, in the validation set, patients were segmented into HRG (n = 21) and LRG (n = 44) in accordance with the optimal threshold value (43.94293). There were also substantial disparities in survival probability between HRG and LRG (*P* = 0.00013) (Figures 3D,E). The AUC value (1-(0.723), 2-(0.746), and 3-(0.638) years) also indicated it performed well in predicting the survival status (Figure 3F). The results as mentioned above demonstrated that the prognostic model exhibited remarkable robustness.

The distribution of clinicopathological features in the two risk groups was demonstrated (Figure 3G). The results of the heatmap showed that the prognostic genes were lowly expressed in the LRG and highly expressed in the HRG; most patients were in Stage II, Stage T3/T4, and N1; the majority of patients were over 40 years old, while there was no obvious distribution pattern in terms of gender.

### 3.4 Differences in enrichment pathways and immune microenvironment were strongly associated with different prognostic populations

**FIGURE 4:**
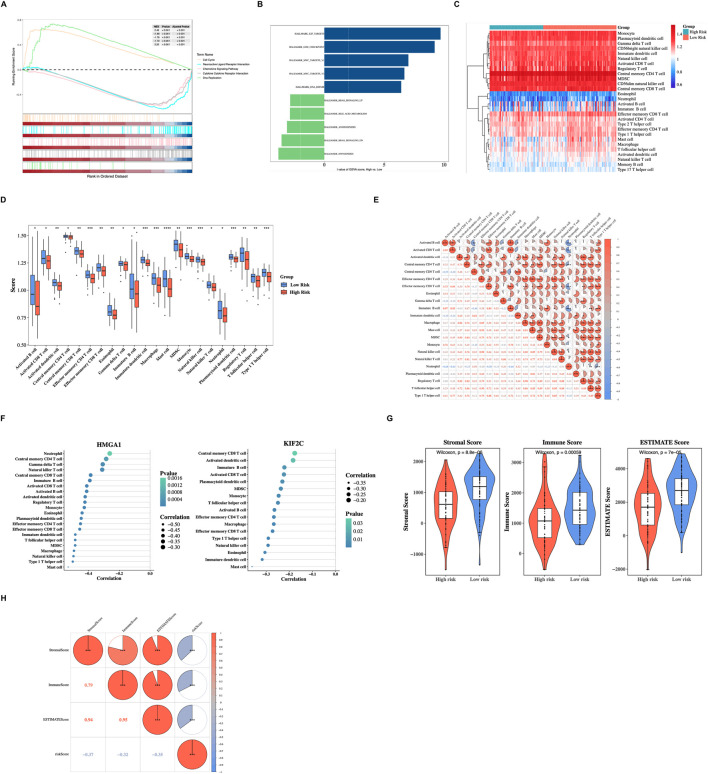
Functional enrichment analysis and immune microenvironment characteristics. **(A)** Gene Set Enrichment Analysis (GSEA) based on KEGG gene sets comparing HRG and LRG. **(B)** Gene Set Variation Analysis (GSVA) showing differentially enriched Hallmark pathways between HRG and LRG. **(C)** Heatmap displaying the infiltration levels of 28 immune cell types in HRG and LRG samples. Color intensity represents the ssGSEA enrichment score for each immune cell type. **(D)** Box plots comparing the infiltration levels of 22 significantly different immune cell types between HRG and LRG. Most immune cells showed lower infiltration scores in HRG (**P < 0.01, Wilcoxon test). **(E)** Correlation heatmap showing the relationships among differentially infiltrated immune cells. Immature B cells and activated B cells showed the strongest positive correlation (cor = 0.97, *P* < 0.001), while neutrophils and activated B cells showed the strongest negative correlation (cor = −0.68, *P* < 0.001). **(F)** Correlation analysis between prognostic genes (HMGA1 and KIF2C) and immune cells. Both genes showed the strongest negative correlation with mast cells (cor = −0.36 and −0.53 for KIF2C and HMGA1, respectively, *P* < 0.01). **(G)** Violin plot comparing ESTIMATE scores (stromal score, immune score, and ESTIMATE score) between HRG and LRG. All three scores were significantly lower in HRG (***P < 0.001, Wilcoxon test). **(H)** Correlation analysis between the risk score and tumor microenvironment scores (StromalScore, ImmuneScore, and ESTIMATEScore). Red indicates positive correlation, and blue indicates negative correlation. ***P < 0.001.

The GSEA results based on the KEGG gene set, included the following pathways: Cell cycle, Neuroactive ligand receptor interaction, Chemokine signaling pathway, Cytokine-cytokine receptor interaction, and DNA replication (Figure 4A; Supplementary Table S8). The upregulated pathways of GSVA included DNA repair, MYC targets V2, and G2M checkpoint, while the downregulated pathways included Myogenesis, KRAS signaling DN, and Angiogenesis (Figure 4B; Supplementary Table S9). These findings furnished a robust basis for the ongoing investigation of the molecular mechanisms underlying PDAC.

Figure 4C illustrates the infiltration levels of immune cells within the HRG and the LRG. The outcomes showed that 22 types of immune cells had substantial disparities between the HRG and the LRG. The outcomes revealed that most cells had lower scores in the HRG (*P* < 0.01) (Figure 4D). Among them, immature B cells and activated B cells demonstrated the strongest positive correlation (cor = 0.97, *P* < 0.001), while neutrophils and activated B cells demonstrated the strongest negative correlation (cor = −0.68, *P* < 0.001) (Figure 4E). Furthermore, KIF2C and HMGA1 demonstrated the strongest negative relationship with mast cells (cor = −0.36, −0.53, *P* < 0.01) (Figure 4F; Supplementary Table S10). Furthermore, there was a notable disparity in the scores of the immune microenvironment in two risk groups (*P* < 0.001) (Figure 4G). Moreover, there was a significant negative correlation between the risk score and the ESTIMATE score, immune score, and stromal score (cor = −0.35, −0.32, −0.37, *P* < 0.001) (Figure 4H). The above outcomes suggested that prognostic genes might influence the immune cell infiltration of PDAC, and this might provide a reference for the clinical management of PDAC.

The lactylation-associated score was significantly higher in the low-risk group than in the high-risk group, indicating a distinct lactylation-related metabolic context between risk subgroups (Supplementary Figure S2A). Moreover, correlation analysis showed that HMGA1 expression was negatively correlated with the lactylation-associated score (r = −0.38, *P* < 0.001) (Supplementary Figure S2B).

### 3.5 The patients' sensitivity to drugs and gene mutation landscape affected the treatment of PDAC patients

**FIGURE 5:**
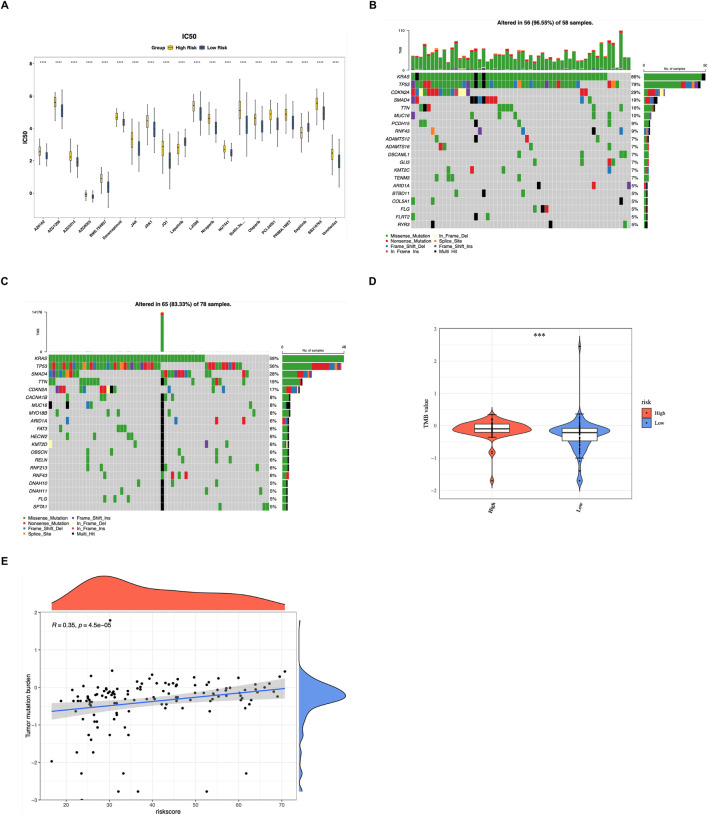
Drug sensitivity analysis and mutation landscape. **(A)** Box plots showing the top 20 drugs with significant differences in predicted IC_50_ values between HRG and LRG. Lower IC_50_ values indicate higher drug sensitivity. Sapitinib and Lapatinib were more effective in HRG, while AZ6102 and Doramapimod were more effective in LRG (****P < 0.0001, Wilcoxon test). **(B,C)** Waterfall plots displaying the top 20 genes with the highest mutation frequencies in HRG **(B)** and LRG **(C)**. **(D)** Violin plot comparing tumor mutational burden (TMB) scores between HRG and LRG. TMB was significantly higher in HRG (***P < 0.001, Wilcoxon test). **(E)** Scatter plot showing the positive correlation between risk scores and TMB scores (cor = 0.35, *P* = 4.5e-05, Spearman correlation).

The lower IC_50_ values indicated that the drugs could achieve a significant therapeutic effect at a lower dose and reduce toxicity and side effects. A total of 127 different drugs were identified (*P* < 0.05) (Supplementary Table S11). Among the top 20 drugs with significant differences, Sapitinib and Lapatinib were more effective in the HRG, while AZ6102 and Doramapimod were more effective in the LRG (*P* < 0.0001) (Figure 5A).

The top 20 genes exhibiting the highest mutation frequencies in the HRG and LRG were presented (Figures 5B,C). The obtained results clearly demonstrated that the missense mutations emerged as the most frequently occurring mutation type. Notably, the mutation rates of KRAS and TP53 not only surpassed 50% in both the HRG and LRG, but also stood out as the most common mutations. This indicated their significant role and potential influence in the genetic alterations associated with these groups, potentially affecting various biological processes and disease progression. Furthermore, there was a marked discrepancy in TMB was observed between the HRG and LRG (*P* < 0.001), and TMB exhibited a notable positive relationship with risk scores (cor = 0.35, *P* = 4.5e-05) (Figures 5D,E). This provided a strong basis for clinicians to formulate personalized treatment plans for patients with different risk stratifications.

### 3.6 The prognostic genes located on autosomes showed significant differences in expression between tumor samples and control samples

**FIGURE 6:**
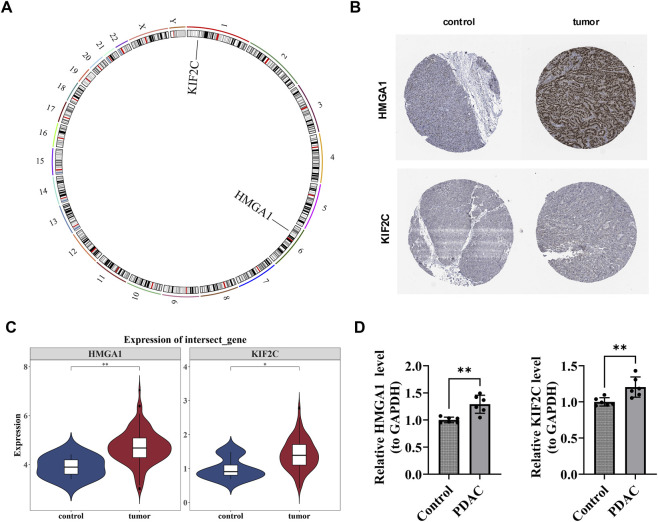
Chromosomal localization and expression validation of prognostic genes in PDAC. **(A)** Chromosomal localization map showing the distribution of prognostic genes on autosomes. KIF2C is located on chromosome 1, and HMGA1 is located on chromosome 6. **(B)** Immunohistochemical analysis of prognostic genes. **(C)** Expression analysis in the training set demonstrating upregulated expression of HMGA1 and KIF2C in PDAC patients compared to control samples. **(D)** RT-qPCR validation showed significant overexpression of HMGA1 and KIF2C in PDAC samples compared with normal controls (*P* < 0.01), confirming the reliability of the bioinformatics analysis results.

The chromosomal localization map revealed that two prognostic genes were located on autosomes: KIF2C on chromosome one and HMGA1 on chromosome 6 (Figure 6A). Both the immunohistochemical analysis of prognostic genes and the expression analysis of prognostic genes in the training set showed that HMGA1 and KIF2C were upregulated in PDAC patients (*P* < 0.05) (Figures 6B,C). The RT-qPCR results showed that HMGA1 and KIF2C were still prominently overexpressed in PDAC samples (*P* < 0.01) (Figure 6D). This was in accordance with the outcomes of the bioinformatics analysis, indicating that the results of the bioinformatics analysis were reliable.

## 4 Discussion

PDAC remains one of the most lethal malignancies, typically characterized by an immunosuppressive tumor microenvironment, in which lactate-driven lactylation modification can promote immune evasion by regulating myeloid-derived suppressor cells (Saloman et al., 2016; Peng et al., 2024). Recent studies indicate that programmed cell death pathways are closely linked to metabolic reprogramming in PDAC (Chen et al., 2021; Zhang et al., 2025). Still, the mechanistic interactions between lactylation and programmed cell death remain urgently in need of exploration. This study integrates multi-dimensional bioinformatics analyses of TCGA and GEO databases to systematically identify two prognostic genes, HMGA1 and KIF2C, closely associated with lactylation and programmed cell death. It constructs a robust risk-stratification model and elucidates their roles in immune microenvironment remodeling, functional pathway regulation, and drug sensitivity, thereby providing new insights for precision medicine in PDAC.

This study identified HMGA1 and KIF2C as genes significantly upregulated in PDAC with crucial prognostic significance. HMGA1 encodes a non-histone chromosomal structural protein belonging to the high-mobility group protein A family, which is overexpressed in most malignancies and associated with tumor invasiveness, chemoresistance, and therapeutic response (Wang L. et al., 2022). Functionally, HMGA1 coordinates oncogenic programs through multiple mechanisms: it promotes lipid synthesis in colorectal cancer (Zhao et al., 2024), enhances the efficacy of palbociclib via PI3K/mTOR signaling in intrahepatic cholangiocarcinoma (Li Z. et al., 2023), and upregulates the pentose phosphate pathway in esophageal squamous cell carcinoma (Liu et al., 2024). Crucially, in PDAC, HMGA1 directly binds the FGF19 promoter and recruits histone-activating modifiers, thereby promoting FGF19 expression, which, in turn, drives autocrine and paracrine tumor growth (Chia et al., 2023). The latest evidence further indicates that elevated HMGA1 is associated with poor prognosis and plays an immunosuppressive role in the tumor microenvironment (Song et al., 2025), and single-cell analysis identifies HMGA1 as a marker of a subpopulation of metastatic cancer cells (Yang D. et al., 2023). Our study expands this understanding by demonstrating that HMGA1 is co-expressed with lactylation-related genes and negatively correlated with mast cell infiltration, suggesting its potential role in lactylation-mediated immune evasion. The consistency between our bioinformatics predictions and experimental verification results highlights the robustness of the findings and positions HMGA1 as a promising therapeutic target.

Similarly, KIF2C, a member of the kinesin superfamily, encodes a key regulator of spindle dynamics and chromosome segregation during mitosis, playing an essential role in maintaining genomic stability (Kreis et al., 2024). The dysregulation of KIF2C is closely associated with tumor progression; its elevated expression can promote tumor cell migration, invasion, and chemoresistance, and impair DNA damage repair (Li R.-Q. et al., 2024). In clear cell renal cell carcinoma, KIF2C exacerbates tumor grade, stage, metastasis status, and a poor prognosis by activating the JAK2/STAT3 signaling axis (Deng et al., 2023). Importantly, KIF2C is significantly overexpressed in PDAC tissues and cell lines (ASPC-1, MIA-PACA-2), driving tumor growth and metastasis (Huang et al., 2023), and its expression level is negatively correlated with patient survival rate (Xiong et al., 2022). This study validated these findings, confirming that KIF2C expression is low in normal pancreatic exocrine cells and undetectable in endocrine cells, but significantly upregulated in PDAC. Notably, KIF2C expression is strongly negatively correlated with mast cell infiltration and associated with the enrichment of cell cycle and G2/M checkpoint pathways in high-risk patients, consistent with its established roles in mitotic regulation and genomic instability. These findings indicate that KIF2C is a key driver of PDAC invasiveness and a potential biomarker for risk stratification.

Functional enrichment analysis revealed that high-risk patients were characterized by abnormal activation of cell cycle and G2/M checkpoint pathways, which was not only associated with lower recurrence-free survival and overall survival, but also predicted enhanced sensitivity to adjuvant chemotherapy and potential resistance to immune checkpoint inhibitors (Mao et al., 2022). This finding is consistent with the established roles of HMGA1 and KIF2C in promoting mitotic progression and genomic instability. In addition to cell cycle dysregulation, our GSEA results also highlighted the enrichment of neuroactive ligand-receptor interaction pathways, reflecting the critical role of neural infiltration in PDAC metastasis. Tumor cells promote distant organ dissemination by utilizing neural pathways (Guo et al., 2023; Bhattacharjee and Ghosh, 2025). Furthermore, upregulation of G2/M checkpoint components (including PLK1) is associated with MEK inhibitor resistance (Zhao et al., 2025), suggesting a potential therapeutic vulnerability in high-risk patients. Notably, PDAC progression is also closely related to angiogenesis; tumor-associated macrophages (TAMs) can promote increased vascular density (Yang et al., 2021). These pathway alterations collectively reveal a synergistic oncogenic program driven by HMGA1 and KIF2C, integrating metabolic reprogramming, cell-cycle dysregulation, and immune-escape mechanisms.

The PDAC tumor microenvironment plays a critical role in disease progression, and the pattern of immune cell infiltration significantly impacts clinical outcomes (Werba et al., 2023). Our analysis revealed 22 types of differentially infiltrated immune cells, among which the immune cell infiltration score in the high-risk group was significantly lower. High B cell infiltration is associated with improved PDAC survival (Delvecchio et al., 2022); we observed a strong positive correlation between immature and activated B cells, suggesting a coordinated adaptive immune response. Conversely, N2 polarized neutrophils, as the dominant population in the PDAC microenvironment, drive tumor progression by promoting proliferation, angiogenesis, tissue remodeling, and immune suppression (Mantovani et al., 2021). Neutrophils undergo a transition from TAN-3 to TAN-0 phenotype during transendothelial migration into the tumor (Wang L. et al., 2023) and enhance metastatic ability by secreting TNF-α and TGF-β1 (Lianyuan et al., 2020). We observed a strong negative correlation between neutrophils and activated B cells, reflecting the antagonistic effect between innate and adaptive immunity in PDAC. Notably, both HMGA1 and KIF2C showed the strongest negative correlation with mast cell infiltration. Mast cells in the PDAC tumor microenvironment have been confirmed to promote immune suppression and fibrous stromal hyperplasia (Ma et al., 2024). In melanoma, mast cells hinder tumor cell homing and metastasis by inhibiting HMGA1 secretion from tumor cells (Benito-Martin et al., 2023), suggesting a potential regulatory axis. Our study proposes that tumors with high HMGA1/KIF2C expression may be associated with reduced mast cell infiltration or altered mast cell functional states, potentially influencing immune surveillance. This requires further verification through co-culture systems, flow cytometry, or spatial transcriptomics to elucidate the causal relationship and therapeutic implications.

In summary, this study successfully identified HMGA1 and KIF2C as prognostic genes and established a robust risk-stratification model integrating lactylation and programmed cell death. Through multi-dimensional analysis, the roles of these two genes in cell cycle regulation and immune microenvironment remodeling were elucidated, providing new insights into the mechanism of PDAC progression. However, several limitations still need attention. First, the conclusions of this study are based on retrospective analysis of public databases, lacking validation from prospective cohorts or functional experiments. Subsequent studies should combine *in vitro* and *in vivo* models to dissect the causal relationship of HMGA1 and KIF2C in lactylation-mediated tumorigenesis and programmed cell death evasion. Second, although the bioinformatics predictions are consistent with the published literature, the causal relationship between HMGA1/KIF2C and specific immune cell subsets (such as mast cells and neutrophils) still needs verification using flow cytometry, co-culture systems, or spatial transcriptomics. Furthermore, the molecular bridge connecting lactylation modification and programmed cell death pathways remains unclear. Integrating proteomics with CRISPR-based functional screening will help clarify the lactylation substrates and programmed cell death regulatory factors regulated by HMGA1 and KIF2C. Finally, the clinical application value of this model needs validation in independent multicenter cohorts, and its predictive value for immune therapy and targeted therapy responses awaits prospective evaluation. At the same time, the number of clinical samples used for qRT-PCR validation in this study was relatively limited. Future studies should expand the sample amount to further improve statistical power and verify the robustness of the results. It should be noted that this study primarily relied on transcriptomic data analysis and has not yet obtained proteomic evidence, such as identification of specific lactation sites or quantitative detection of lactation levels, to directly verify the lactation modification of HMGA1 and KIF2C. Therefore, future studies should combine proteomic analysis or related experiments to further confirm the direct lactation mechanism.

## 5 Conclusion

In summary, this study not only systematically integrated the lactylation and programmed cell death-associated genes HMGA1 and KIF2C into a PDAC prognostic evaluation system for the first time but also revealed their potential immune regulatory and pathway interaction mechanisms. These findings provide new insights into the precise typing, prognostic assessment, and development of targeted immune combination therapy strategies for PDAC.

## Data Availability

The original contributions presented in the study are included in the article/Supplementary Material, further inquiries can be directed to the corresponding authors.

## References

[B1] Al-Rawi N. H. Rizvi Z. Mkadmi S. Abu Kou R. Elmabrouk N. Alrashdan M. S. (2023). Differential expression profile of salivary oncomiRNAs among smokeless tobacco users. Eur. J. Dent. 17, 1215–1220. 10.1055/s-0043-1761191 36812928 PMC10756836

[B2] Benito-Martin A. Nogués L. Hergueta-Redondo M. Castellano-Sanz E. Garvin E. Cioffi M. (2023). Mast cells impair melanoma cell homing and metastasis by inhibiting HMGA1 secretion. Immunology 168, 362–373. 10.1111/imm.13604 36352838

[B3] Bhattacharjee K. Ghosh A. (2025). Identification of key regulators in pancreatic ductal adenocarcinoma using network theoretical approach. PLoS One 20, e0313738. 10.1371/journal.pone.0313738 39869563 PMC11771905

[B4] Chen H. Boutros P. C. (2011). VennDiagram: a package for the generation of highly-customizable venn and Euler diagrams in R. BMC Bioinforma. 12, 35. 10.1186/1471-2105-12-35 21269502 PMC3041657

[B5] Chen X. Zeh H. J. Kang R. Kroemer G. Tang D. (2021). Cell death in pancreatic cancer: from pathogenesis to therapy. Nat. Rev. Gastroenterol. Hepatol. 18, 804–823. 10.1038/s41575-021-00486-6 34331036

[B6] Chia L. Wang B. Kim J.-H. Luo L. Z. Shuai S. Herrera I. (2023). HMGA1 induces FGF19 to drive pancreatic carcinogenesis and stroma formation. J. Clin. Invest. 133, e151601. 10.1172/JCI151601 36919699 PMC10014113

[B7] Connor A. A. Gallinger S. (2022). Pancreatic cancer evolution and heterogeneity: integrating omics and clinical data. Nat. Rev. Cancer 22, 131–142. 10.1038/s41568-021-00418-1 34789870

[B8] Delvecchio F. R. Goulart M. R. Fincham R. E. A. Bombadieri M. Kocher H. M. (2022). B cells in pancreatic cancer stroma. World J. Gastroenterol. 28, 1088–1101. 10.3748/wjg.v28.i11.1088 35431504 PMC8985484

[B9] Deng H. Gong X. Ji G. Li C. Cheng S. (2023). KIF2C promotes clear cell renal cell carcinoma progression *via* activating JAK2/STAT3 signaling pathway. Mol. Cell. Probes 72, 101938. 10.1016/j.mcp.2023.101938 37863123

[B10] Emura T. Matsui S. Chen H.-Y. (2019). compound.Cox: univariate feature selection and compound covariate for predicting survival. Comput. Methods Programs Biomed. 168, 21–37. 10.1016/j.cmpb.2018.10.020 30527130

[B11] Garajová I. Peroni M. Gelsomino F. Leonardi F. (2023). A simple overview of pancreatic cancer treatment for clinical oncologists. Curr. Oncol. 30, 9587–9601. 10.3390/curroncol30110694 37999114 PMC10669959

[B12] Guo K. Zhao Y. Cao Y. Li Y. Yang M. Tian Y. (2023). Exploring the key genetic association between chronic pancreatitis and pancreatic ductal adenocarcinoma through integrated bioinformatics. Front. Genet. 14, 1115660. 10.3389/fgene.2023.1115660 37501719 PMC10369079

[B13] Gustavsson E. K. Zhang D. Reynolds R. H. Garcia-Ruiz S. Ryten M. (2022). Ggtranscript: an R package for the visualization and interpretation of transcript isoforms using ggplot2. Bioinformatics 38, 3844–3846. 10.1093/bioinformatics/btac409 35751589 PMC9344834

[B14] Hänzelmann S. Castelo R. Guinney J. (2013). GSVA: gene set variation analysis for microarray and RNA-seq data. BMC Bioinforma. 14, 7. 10.1186/1471-2105-14-7 23323831 PMC3618321

[B15] Hsu S.-K. Jadhao M. Liao W.-T. Chang W.-T. Hung C.-T. Chiu C.-C. (2022). Culprits of PDAC resistance to gemcitabine and immune checkpoint inhibitor: tumour microenvironment components. Front. Mol. Biosci. 9, 1020888. 10.3389/fmolb.2022.1020888 36299300 PMC9589289

[B16] Hu Y. Yan C. Hsu C.-H. Chen Q.-R. Niu K. Komatsoulis G. A. (2014). OmicCircos: a simple-to-use R package for the circular visualization of multidimensional omics data. Cancer Inf. 13, 13–20. 10.4137/CIN.S13495 24526832 PMC3921174

[B17] Huang X. Zhao F. Wu Q. Wang Z. Ren H. Zhang Q. (2023). KIF2C facilitates tumor growth and metastasis in pancreatic ductal adenocarcinoma. Cancers (Basel) 15, 1502. 10.3390/cancers15051502 36900292 PMC10000478

[B18] Ishwaran H. Kogalur U. B. Blackstone E. H. Lauer M. S. (2008). Random survival forests. Ann. Appl. Stat. 2, 841–860. 10.1214/08-AOAS169

[B19] Jentzsch V. Davis J. A. A. Djamgoz M. B. A. (2020). Pancreatic cancer (PDAC): introduction of evidence-based complementary measures into integrative clinical management. Cancers (Basel) 12. 10.3390/cancers12113096 33114159 PMC7690843

[B20] Jiang F. Zhou H. Shen H. (2022). Identification of critical biomarkers and immune infiltration in Rheumatoid arthritis based on WGCNA and LASSO algorithm. Front. Immunol. 13, 925695. 10.3389/fimmu.2022.925695 35844557 PMC9277141

[B21] Kreis N.-N. Moon H. H. Wordeman L. Louwen F. Solbach C. Yuan J. (2024). KIF2C/MCAK a prognostic biomarker and its oncogenic potential in malignant progression, and prognosis of cancer patients: a systematic review and meta-analysis as biomarker. Crit. Rev. Clin. Lab. Sci. 61, 404–434. 10.1080/10408363.2024.2309933 38344808 PMC11815995

[B22] Kumar M. Sonker P. K. Saroj A. Jain A. Bhattacharjee A. Saroj R. K. (2020). Parametric survival analysis using R: illustration with lung cancer data. Cancer Rep. Hob. 3, e1210. 10.1002/cnr2.1210 32794636 PMC7941555

[B23] Li L. Sun S. Wu Y. Lu J. He J. Chen K. (2023a). Lactate and protein lactylation: the ugly duckling of energy as the sculpture artist of proteins. Sci. Bull. (Beijing). 68, 2510–2514. 10.1016/j.scib.2023.09.038 37806798

[B24] Li Z. Zhou H. Xia Z. Xia T. Du G. Franziska S. D. (2023b). HMGA1 augments palbociclib efficacy *via* PI3K/mTOR signaling in intrahepatic cholangiocarcinoma. Biomark. Res. 11, 33. 10.1186/s40364-023-00473-w 36978140 PMC10053751

[B25] Li F. Si W. Xia L. Yin D. Wei T. Tao M. (2024a). Positive feedback regulation between glycolysis and histone lactylation drives oncogenesis in pancreatic ductal adenocarcinoma. Mol. Cancer 23, 90. 10.1186/s12943-024-02008-9 38711083 PMC11071201

[B26] Li G. Liao C. Chen J. Wang Z. Zhu S. Lai J. (2024b). Targeting the MCP-GPX4/HMGB1 axis for effectively triggering immunogenic ferroptosis in pancreatic ductal adenocarcinoma. Adv. Sci. (Weinh) 11, e2308208. 10.1002/advs.202308208 38593415 PMC11151063

[B27] Li R.-Q. Yang Y. Qiao L. Yang L. Shen D.-D. Zhao X.-J. (2024c). KIF2C: an important factor involved in signaling pathways, immune infiltration, and DNA damage repair in tumorigenesis. Biomed. Pharmacother. 171, 116173. 10.1016/j.biopha.2024.116173 38237349

[B28] Lianyuan T. Gang L. Ming T. Dianrong X. Chunhui Y. Zhaolai M. (2020). Tumor associated neutrophils promote the metastasis of pancreatic ductal adenocarcinoma. Cancer Biol. Ther. 21, 937–945. 10.1080/15384047.2020.1807250 32835587 PMC7583704

[B29] Liu M.-J. Zhao Y. Li Q.-T. Lei X.-Y. He K.-Y. Guo J.-R. (2024). HMGA1 promotes the progression of esophageal squamous cell carcinoma by elevating TKT-mediated upregulation of pentose phosphate pathway. Cell Death Dis. 15, 541. 10.1038/s41419-024-06933-x 39080260 PMC11289123

[B30] Love M. I. Huber W. Anders S. (2014). Moderated estimation of fold change and dispersion for RNA-seq data with DESeq2. Genome Biol. 15, 550. 10.1186/s13059-014-0550-8 25516281 PMC4302049

[B31] Ma Y. Zhao X. Feng J. Qiu S. Ji B. Huang L. (2024). Tumor-infiltrating mast cells confer resistance to immunotherapy in pancreatic cancer. iScience 27, 111085. 10.1016/j.isci.2024.111085 39473974 PMC11514315

[B32] Mantovani A. Marchesi F. Jaillon S. Garlanda C. Allavena P. (2021). Tumor-associated myeloid cells: diversity and therapeutic targeting. Cell. Mol. Immunol. 18, 566–578. 10.1038/s41423-020-00613-4 33473192 PMC8027665

[B33] Mao Y. Cheng W. Yang Q. Li L. Hu W. Shuang Z. (2022). The enhanced cell cycle related to the response to adjuvant therapy in pancreatic ductal adenocarcinoma. Genomics 114, 95–106. 10.1016/j.ygeno.2021.11.036 34863899

[B34] Mayakonda A. Lin D.-C. Assenov Y. Plass C. Koeffler H. P. (2018). Maftools: efficient and comprehensive analysis of somatic variants in cancer. Genome Res. 28, 1747–1756. 10.1101/gr.239244.118 30341162 PMC6211645

[B35] Mizrahi J. D. Surana R. Valle J. W. Shroff R. T. (2020). Pancreatic cancer. Lancet 395, 2008–2020. 10.1016/S0140-6736(20)30974-0 32593337

[B36] Narayanan S. Vicent S. Ponz-Sarvisé M. (2021). PDAC as an immune evasive disease: can 3D model systems aid to tackle this clinical problem? Front. Cell Dev. Biol. 9, 787249. 10.3389/fcell.2021.787249 34957115 PMC8703167

[B37] Orifjon S. Jammatov J. Sousa C. Barros R. Vasconcelos O. Rodrigues P. (2023). Translation and adaptation of the adult developmental coordination Disorder/Dyspraxia checklist (ADC) into Asian Uzbekistan. Sports (Basel) 11, 135. 10.3390/sports11070135 37505622 PMC10383954

[B38] Peng T. Sun F. Yang J.-C. Cai M.-H. Huai M.-X. Pan J.-X. (2024). Novel lactylation-related signature to predict prognosis for pancreatic adenocarcinoma. World J. Gastroenterol. 30, 2575–2602. 10.3748/wjg.v30.i19.2575 38817665 PMC11135411

[B65] Qin H. Abulaiti A. Maimaiti A. Abulaiti Z. Fan G. Aili Y. (2023). Integrated machine learning survival framework develops a prognostic model based on inter-crosstalk definition of mitochondrial function and cell death patterns in a large multicenter cohort for lower-grade glioma. J. Transl. Med. 21 (1), 588. 10.1186/s12967-023-04468-x 37660060 PMC10474752

[B39] Rabinowitz J. D. Enerbäck S. (2020). Lactate: the ugly duckling of energy metabolism. Nat. Metab. 2, 566–571. 10.1038/s42255-020-0243-4 32694798 PMC7983055

[B40] Rahib L. Smith B. D. Aizenberg R. Rosenzweig A. B. Fleshman J. M. Matrisian L. M. (2014). Projecting cancer incidence and deaths to 2030: the unexpected burden of thyroid, liver, and pancreas cancers in the United States. Cancer Res. 74, 2913–2921. 10.1158/0008-5472.CAN-14-0155 24840647

[B41] Rahib L. Wehner M. R. Matrisian L. M. Nead K. T. (2021). Estimated projection of US cancer incidence and death to 2040. JAMA Netw. Open 4, e214708. 10.1001/jamanetworkopen.2021.4708 33825840 PMC8027914

[B42] Ritchie M. E. Phipson B. Wu D. Hu Y. Law C. W. Shi W. (2015). Limma powers differential expression analyses for RNA-sequencing and microarray studies. Nucleic Acids Res. 43, e47. 10.1093/nar/gkv007 25605792 PMC4402510

[B43] Saloman J. L. Albers K. M. Li D. Hartman D. J. Crawford H. C. Muha E. A. (2016). Ablation of sensory neurons in a genetic model of pancreatic ductal adenocarcinoma slows initiation and progression of cancer. Proc. Natl. Acad. Sci. U. S. A. 113, 3078–3083. 10.1073/pnas.1512603113 26929329 PMC4801275

[B44] Schneider M. Hackert T. Strobel O. Büchler M. W. (2021). Technical advances in surgery for pancreatic cancer. Br. J. Surg. 108, 777–785. 10.1093/bjs/znab133 34046668

[B45] Siegel R. L. Giaquinto A. N. Jemal A. (2024). Cancer statistics, 2024. CA Cancer J. Clin. 74, 12–49. 10.3322/caac.21820 38230766

[B46] Song W. Yu Y. Wang S. Cui Z. Zhu Q. Liu W. (2025). Metabolic reprogramming shapes the immune microenvironment in pancreatic adenocarcinoma: prognostic implications and therapeutic targets. Front. Immunol. 16, 1555287. 10.3389/fimmu.2025.1555287 40191200 PMC11968369

[B47] Wang Y. Patti G. J. (2023). The Warburg effect: a signature of mitochondrial overload. Trends Cell Biol. 33, 1014–1020. 10.1016/j.tcb.2023.03.013 37117116 PMC10600323

[B48] Wang L. Zhang J. Xia M. Liu C. Zu X. Zhong J. (2022a). High Mobility Group A1 (HMGA1): structure, biological function, and therapeutic potential. Int. J. Biol. Sci. 18, 4414–4431. 10.7150/ijbs.72952 35864955 PMC9295051

[B49] Wang Y. He Y. Cao L. Peng X. Gu Z. Yan J. (2022b). Exploring the correlation analysis of immune microenvironment, mutation burden and prognosis of papillary thyroid carcinoma based on Estimate algorithm. Gland. Surg. 11, 860–867. 10.21037/gs-22-211 35694099 PMC9177282

[B50] Wang L. Liu Y. Dai Y. Tang X. Yin T. Wang C. (2023a). Single-cell RNA-seq analysis reveals BHLHE40-driven pro-tumour neutrophils with hyperactivated glycolysis in pancreatic tumour microenvironment. Gut 72, 958–971. 10.1136/gutjnl-2021-326070 35688610 PMC10086491

[B51] Wang Y. Bin T. Tang J. Xu X.-J. Lin C. Lu B. (2023b). Construction of an acute myeloid leukemia prognostic model based on m6A-related efferocytosis-related genes. Front. Immunol. 14, 1268090. 10.3389/fimmu.2023.1268090 38077322 PMC10704160

[B52] Wang Y. Zhuang H. Jiang X.-H. Zou R.-H. Wang H.-Y. Fan Z.-N. (2023c). Unveiling the key genes, environmental toxins, and drug exposures in modulating the severity of ulcerative colitis: a comprehensive analysis. Front. Immunol. 14, 1162458. 10.3389/fimmu.2023.1162458 37539055 PMC10394652

[B53] Werba G. Weissinger D. Kawaler E. A. Zhao E. Kalfakakou D. Dhara S. (2023). Single-cell RNA sequencing reveals the effects of chemotherapy on human pancreatic adenocarcinoma and its tumor microenvironment. Nat. Commun. 14, 797. 10.1038/s41467-023-36296-4 36781852 PMC9925748

[B54] Xiong J. Wu R. He A. Hou P. Wang J. Zhang R. (2022). Comprehensive analysis of the effects of KIF2C on prognosis, biological functions and immune infiltration in PAAD. Tissue Cell 78, 101900. 10.1016/j.tice.2022.101900 36029726

[B55] Yang Y. Guo Z. Chen W. Wang X. Cao M. Han X. (2021). M2 macrophage-derived exosomes promote angiogenesis and growth of pancreatic ductal adenocarcinoma by targeting E2F2. Mol. Ther. 29, 1226–1238. 10.1016/j.ymthe.2020.11.024 33221435 PMC7934635

[B56] Yang D. Moniruzzaman R. Wang H. Wang H. Chen Y. (2023a). Cross-Dataset single-cell analysis identifies temporal alterations in cell populations of primary pancreatic tumor and liver metastasis. Cancers (Basel) 15, 2396. 10.3390/cancers15082396 37190324 PMC10137114

[B57] Yang Z. Su W. Wei X. Qu S. Zhao D. Zhou J. (2023b). HIF-1α drives resistance to ferroptosis in solid tumors by promoting lactate production and activating SLC1A1. Cell Rep. 42, 112945. 10.1016/j.celrep.2023.112945 37542723

[B58] Yu G. Wang L.-G. Han Y. He Q.-Y. (2012). clusterProfiler: an R package for comparing biological themes among gene clusters. OMICS 16, 284–287. 10.1089/omi.2011.0118 22455463 PMC3339379

[B59] Yu Y. Li Y. Zhou L. Cheng X. Gong Z. (2024). Hepatic stellate cells promote hepatocellular carcinoma development by regulating histone lactylation: novel insights from single-cell RNA sequencing and spatial transcriptomics analyses. Cancer Lett. 604, 217243. 10.1016/j.canlet.2024.217243 39260669

[B60] Zhang W. Shan G. Bi G. Hu Z. Yi Y. Zeng D. (2025). Lactylation and regulated cell death. Biochim. Biophys. Acta Mol. Cell Res. 1872, 119927. 10.1016/j.bbamcr.2025.119927 40023198

[B61] Zhao Y. Li M. Yao X. Fei Y. Lin Z. Li Z. (2020). HCAR1/MCT1 regulates tumor ferroptosis through the lactate-mediated AMPK-SCD1 activity and its therapeutic implications. Cell Rep. 33, 108487. 10.1016/j.celrep.2020.108487 33296645

[B62] Zhao Y. Liu M.-J. Zhang L. Yang Q. Sun Q.-H. Guo J.-R. (2024). High mobility group A1 (HMGA1) promotes the tumorigenesis of colorectal cancer by increasing lipid synthesis. Nat. Commun. 15, 9909. 10.1038/s41467-024-54400-0 39548107 PMC11568219

[B63] Zhao B. Fang R. Schürmann H. Hemmer E. J. Mayer G. L. Trajkovic-Arsic M. (2025). PLK1 blockade enhances the anti-tumor effect of MAPK inhibition in pancreatic ductal adenocarcinoma. Cell Rep. 44, 115541. 10.1016/j.celrep.2025.115541 40188436

[B64] Zheng Y. Wen Y. Cao H. Gu Y. Yan L. Wang Y. (2021). Global characterization of immune infiltration in clear cell renal cell carcinoma. Onco. Targets. Ther. 14, 2085–2100. 10.2147/OTT.S282763 33790572 PMC7997590

