# Supplementary figures and images for: Exploring prognostic genes related to lactylation and programmed cell death in pancreatic ductal adenocarcinoma: a comprehensive study combining bulk transcriptomics and experimental verification
Source: Front Genet. 2026 Mar 12;17:1774953. doi: 10.3389/fgene.2026.1774953 (PMC13017068; doi:10.3389/fgene.2026.1774953)

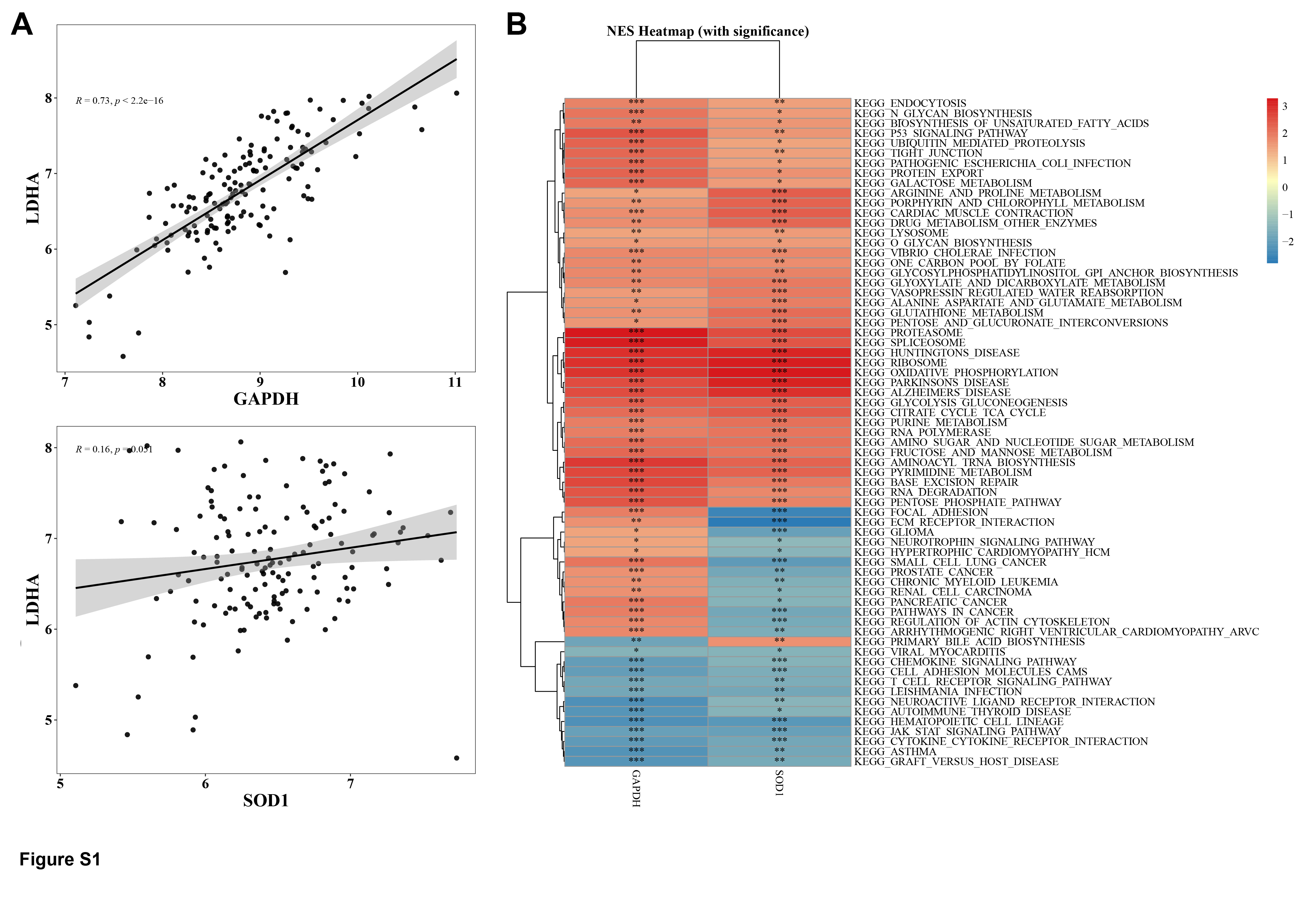

Supplement: Supplementary file 4 [file Image1.jpeg]

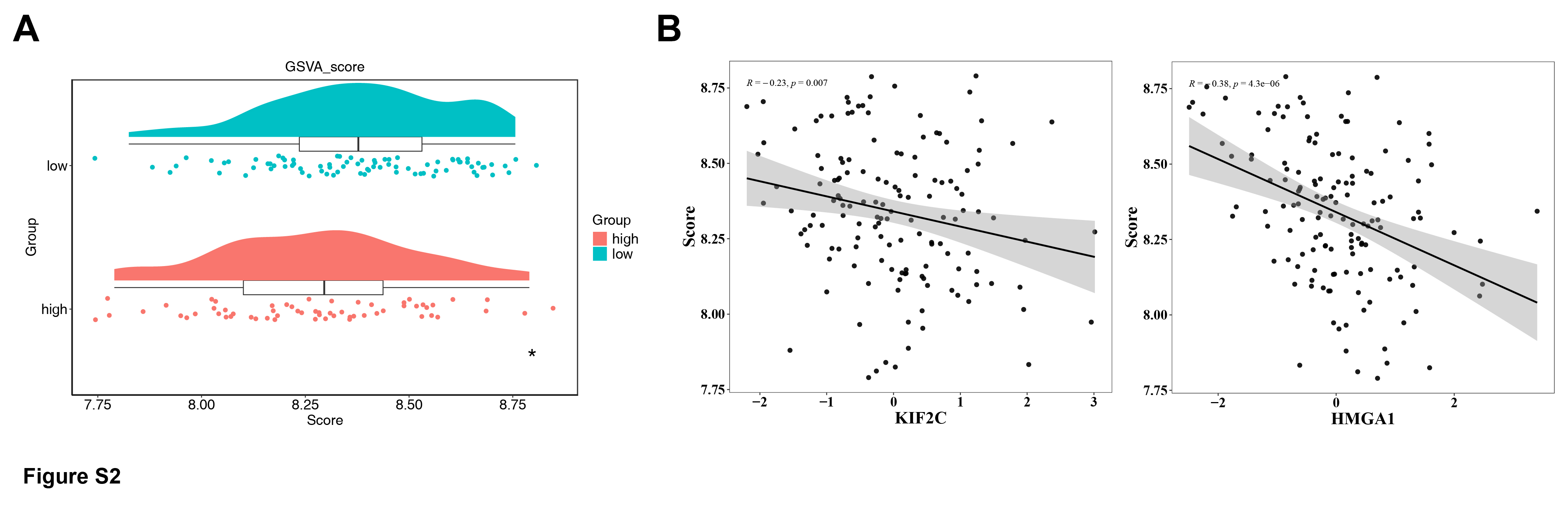

Supplement: Supplementary file 5 [file Image2.jpeg]
